# Impact of metal vs non-absorbable, polymer clips during laparoscopic cholecystectomy

**DOI:** 10.1007/s00464-025-11559-x

**Published:** 2025-02-12

**Authors:** A. J. Bartholomew, C. Jing, K. P. Economopoulos, A. Sizemore, J. Lim, S. Record, S. Greene, J. M. Ladowski, T. C. Howell, A. Gordee, M. Kuchibhtala, J. Yoo, K. Jain-Spangler, A. D. Michaels, P. A. Fong, J. A. Greenberg, K. A. Seymour

**Affiliations:** 1 Department of Surgery, Duke University School of Medicine, Durham, NC USA; 2 Duke University School of Medicine, Durham, NC USA; 3 Department of Biostatistics and Bioinformatics, School of Medicine, Duke University, Durham, NC USA; 4 Duke University Health System, 407 Crutchfield Street, Durham, NC 27704 USA

**Keywords:** Cholecystectomy, Clip type, Metal clip, Polymer clip, Safety, Cost

## Abstract

**Background:**

Titanium metal clips have classically been used to occlude the cystic artery and duct during laparoscopic cholecystectomy (LC). Non-absorbable, polymer clips are an alternative with a locking feature. There is limited research evaluating the adoption, safety, and cost of these clips during cholecystectomy.

**Methods:**

A retrospective review was conducted on patients undergoing elective LC from 2017 to 2019. The cohort was divided based on the use of metal or polymer clips. The primary outcome was 30-day emergency department (ED) visit rate. Secondary outcomes included readmission and complications. Surgeon utilization and cost comparison were assessed. Chi square, Wilcoxon rank-sum, and multivariable logistic regression was performed.

**Results:**

1244 patients underwent LC by 38 surgeons, of which 934 (75.1%) utilized metal clips. Thirty-day ED presentation was 8.5%, with a higher rate for the polymer clip group (12.4% vs 7.2%, p = 0.005); 79% of presentations were related to the operation. On adjusted analysis, ED visits were associated with hospital facility and insurance payor. Thirty-day readmission rate was comparable for polymer and metal clips (4.9% vs 3.2%, p = 0.18, respectively). Most surgeons used metal clips (58%) and there was no impact based on fellowship training. Those who preferentially utilized polymer clips had more recently graduated from medical school (p = 0.02) and were more likely to perform intraoperative cholangiograms (p < 0.001). The device cost difference favored polymer clips by $75 per case.

**Conclusion:**

Polymer clips are a safe alternative to metal clips, with a similarly low complication profile. Despite an increase in 30-day ED visit rate in the polymer group, adjusted analysis demonstrated an association with hospital facility and insurance type, and not clip type. Given LC is one of the most commonly performed operations worldwide, the benefit of locking polymer clips should be incorporated into intraoperative decision making.

**Supplementary Information:**

The online version contains supplementary material available at 10.1007/s00464-025-11559-x.

Laparoscopic cholecystectomy (LC) is one of the most frequently performed abdominal surgeries worldwide, with approximately 750,000 cases annually in the United States alone [1, 2]. While the common goal in this operation is to obtain the critical view of safety prior to ligating the cystic duct and artery, the method in which these structures are secured is largely surgeon-dependent [3]. Classically, this has been accomplished with metal clips, though there is work describing a variety of techniques including suturing, stapling, and various energy devices [4–7]. Failure to properly secure the cystic duct can result in bile leak, complicating approximately 1% of cholecystectomies, has been associated with increased morbidity and mortality [8, 9].

Recently, non-absorbable polymer clips have increased in popularity and differ from traditional metal clips. Polymer clips are easy to use, radiolucent, and inert; the locking mechanism provides haptic feedback during occlusion of the cystic duct. The use of non-absorbable polymer clips is clinically common but supported by low-level data with a lack of randomized trials. A recent study compared non-absorbable polymer clips to traditional metal clips during LC. The polymer clips demonstrated superior performance and intraoperative efficacy of securing of the cystic ducts larger than 4 mm in diameter [5]. However, minimal work has been done comparing postoperative complications and re-presentations to care following LC.

Emergency department (ED) visits after cholecystectomy provide care for surgical and non-surgical complications, along with preventable, non-urgent issues. The rate of 30-day ED visits following LC is more than double the readmission rate [10]. Therefore, we sought to examine whether there were differences in 30-day ED visits based on clip type. We hypothesized that there would be no difference in ED utilization based on clip type.

## Methods

### Cohort

**Fig. 1:**
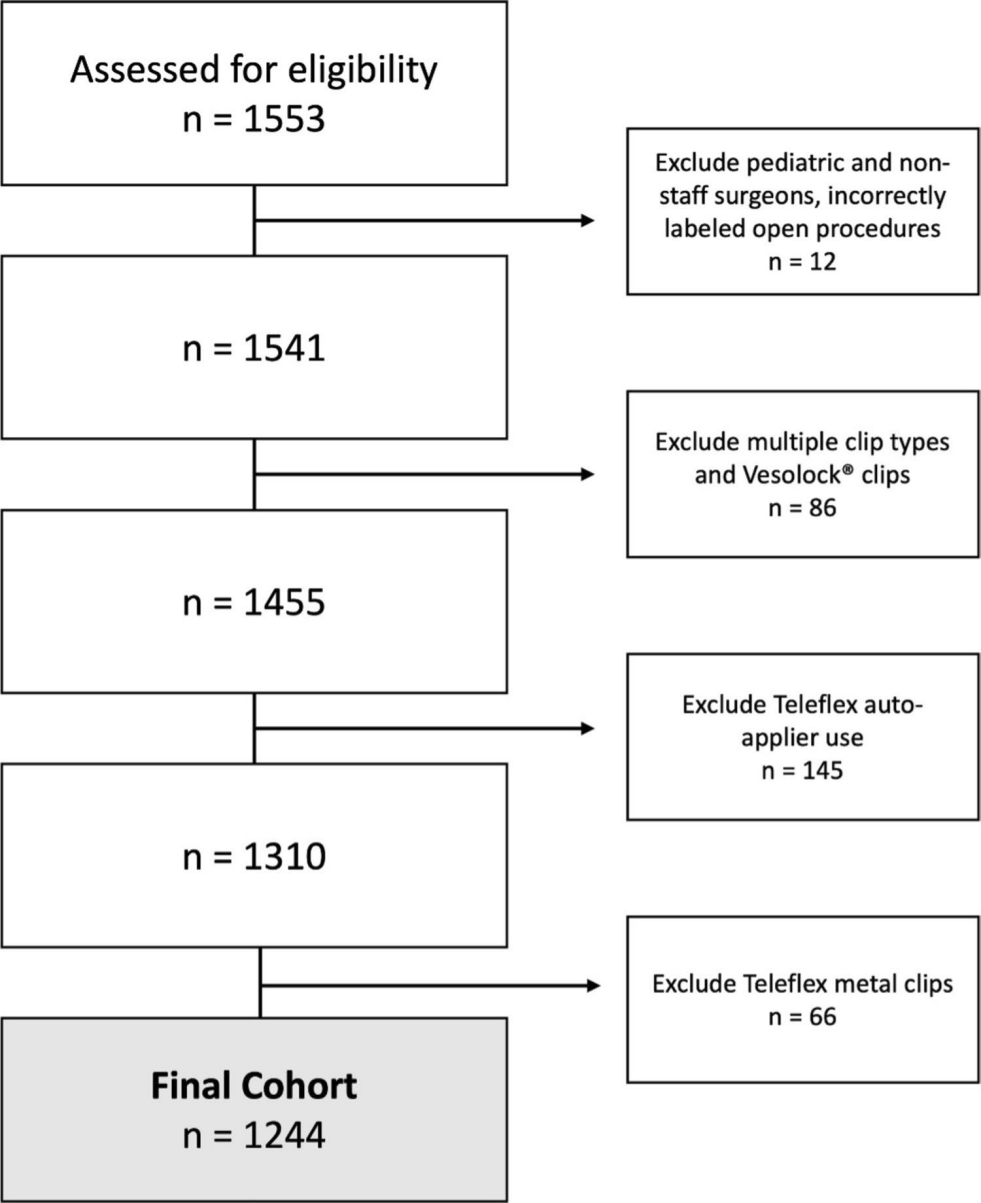
Consort diagram demonstrating exclusion criteria and final cohort size

All patients greater than 18 years who underwent elective LC (current procedural terminology codes 45,762 and 45,763) from January 1, 2017, to December 31, 2019 within a single healthcare system were included in the initial cohort (Fig. 1). Patients were excluded when additional planned procedures were performed, excluding esophagogastroduodenoscopy or liver biopsy. Over 1553 cases were reviewed for eligibility. Exclusion criteria included pregnant patients, robotic approach, cases with operative time longer than five hours, cases performed by pediatric surgeons or non-faculty surgeons, cases with multiple clip types within a single operation, and cases with inaccurate data capture (ex. clips used in open procedures, robotic clip appliers, investigational clips, and discontinued clip appliers).

### Variable definitions

Chart abstraction was performed to identify patients including age, race, ethnicity, body mass index (BMI), payor status, comorbidities including hypertension, hyperlipidemia, depression, chronic kidney disease (CKD), cardiac disease (history of myocardial infarction, percutaneous coronary intervention, or cardiac surgery), gastroesophageal reflux disease (GERD), active anticoagulant use, and obstructive sleep apnea. Patients were binned by number of comorbidities into three groups: 0, 1, or ≥ 2 comorbidities. Additional details included whether a patient underwent endoscopic retrograde pancreatography (ERCP). Operative characteristics included location (the health system has one academic hospital, two community hospitals, and multiple ambulatory surgery centers), operative time, participation of a trainee, use of intraoperative cholangiogram (IOC), and demographics of the primary surgeon such as specialty, years since medical school graduation, and fellowship completion. Insurance status was grouped as commercial, Medicaid, Medicare, or other (uninsured, other government insurance, self-pay, or special programs). All postoperative outcomes were assessed from the date of index operation through 30 calendar days. Individual presentations to the ED were categorized as related or unrelated to the index operation based on modified Goldfield criteria [11, 12].

### Clip type and associated cost data

**Fig. 2:**
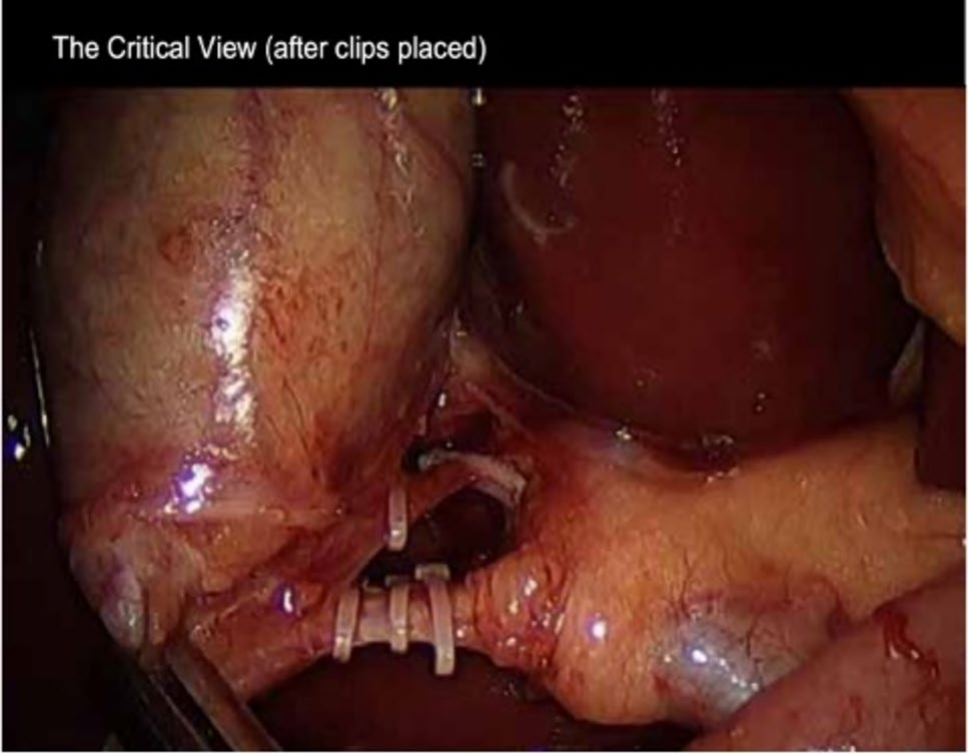
Representative intraoperative image demonstrating use of non-absorbing clips on the cystic duct and artery after obtaining the critical view of safety

Clip type and brand were recorded for each case. Polymer clips presented in this study are Hem-o-Lock clips from Weck Surgical Instruments [Teleflex Medical, Durham, NC, USA; Fig. 2]. Metal clips included devices supplied by several manufacturers, including Medtronic, Applied Medical, Ethicon, and Johnson & Johnson. Clip cost was derived from publicly available data by a third-party company [IQVIA Holdings Inc], which generated pricing information from a sample size of 20% of US hospitals and included the years 2018–2022. Cost for clips was inflation-adjusted to 2022 US dollars.

### Statistical analysis

The primary outcome was presentation to the emergency department (ED) within 30 days of the index operation. Secondary outcomes included readmission rate, infection, fluid collection on cross-sectional imaging, and need for ERCP. For all surgeons included in the cohort, a preference for either metal or polymer clips was defined based on > 50% of operations utilizing a specific clip type. Chi square or Fisher’s exact test, where appropriate, and Wilcoxon rank-sum tests were used for univariate associations. Multivariable regression was used for the primary outcome with variables determined a priori including clip type, location of procedure, race/ethnicity, insurance payor status, and number of comorbidities. All statistical analyses were conducted in IBM SPSS® software version 19.0 or higher (IBM Corp, Armonk, NY) and an alpha of 0.05 was considered significant. [13]

## Results

### Patient cohort and operative details

A total of 1244 patients were included in the final cohort with 934 (75.1%) having undergone LC with metal clips and 310 (24.9%) with polymer clips. Among the entire cohort, median age was 50 years [IQR 38.0–63.0], BMI was 30.4 kg/m^2^ [26.0 kg/m^2^–36.2 kg/m^2^], 74.5% were female, and the majority were white (66.6%). Within the polymer group, there was a significantly higher BMI (32.1 kg/m^2^ vs 30.0 kg/m^2^, *p* = 0.001), but no overall baseline differences in comorbidities or anticoagulant use (Table 1).

**Table 1:** Patient characteristics stratified by clip type

Variable	Total	Metal clip	Polymer clip	*p*
	N = 1244	N = 934	N = 310	
Age (years)	50 [38–63]	51 [38–63]	49 [38–62]	0.19
Female	927 (74.5)	698 (74.7)	229 (73.9)	0.76
White	828 (66.6)	649 (69.5)	179 (57.7)	0.003
Black	274 (22.0)	180 (19.3)	94 (30.3)	
Asian	34 (2.7)	25 (2.7)	9 (2.9)	
Other	79 (6.4)	59 (6.3)	13 (4.2)	
Not reported or declined	29 (2.3)	21 (2.2)	15 (4.8)	
Hispanic	88 (7.1)	67 (7.2)	21 (6.8)	0.032
BMI kg/m^2^	30.4 [26.0–36.2]	30.0 [25.7 –35.5]	32.1 [27.1–37.9]	0.001
Comorbidities				
Hypertension	486 (39.2)	353 (37.8)	133 (43.3)	0.085
Chronic kidney disease	56 (4.5)	42 (4.5)	14 (4.6)	0.962
Cardiac disease	156 (12.6)	119 (12.7)	37 (12.1)	0.752
Depression	191 (15.4)	141 (15.1)	50 (16.3)	0.616
Obstructive sleep apnea	136 (11.0)	93 (10.0)	43 (14.0)	0.049
Comorbidities, number				< 0.001
0	353 (28.4)	272 (29.1)	81 (26.4)	
1	345 (27.8)	256 (27.4)	89 (29.0)	
2+	543 (43.8)	406 (43.5)	137 (44.6)	
Anticoagulant use	251 (20.2)	183 (19.6)	68 (22.1)	0.333
Insurance				0.83
Commercial	788 (63.3)	596 (63.8)	192 (61.9)	
Medicare/Medicaid	397 (31.9)	296 (31.7)	101 (32.6)	
Other	32 (2.6)	22 (2.4)	10 (3.2)	
Self-pay	27 (2.2)	20 (2.1)	7 (2.3)	

Values represented as n (%) or median [IQR]

*BMI* body mass index, *SD* standard deviation, *IQR* interquartile range p < 0.05

The majority of cases were performed in a community hospital (76.5%), 22.3% were performed at an academic hospital, and 1.1% were performed at an ambulatory surgery center. Median operative time was 54 min [34–80] with a significantly longer time (67.5 vs. 47.0 min, *p* < 0.001, Table 2) for polymer cases; this group had a lower rate of intraoperative cholangiograms (3.21% vs 0.32%, *p* < 0.003) and higher rates of trainee participation (83.0% vs. 67.3%, *p* < 0.001). There were no conversions from a laparoscopic to an open approach.

**Table 2:** Operative characteristics by clip type with procedure and surgeon level data

Variable	Total	Metal clip	Polymer clip	*p*
Operative details				
N (%)	1244 (100)	934 (75.1)	310 (24.9)	
Trainee participation	887 (71.3)	626 (67.0)	261 (84.2)	< 0.001
Operative time (minutes)	54 [34–80]	47 [30–77]	67.5 [51–84]	< 0.001
;Intraoperative cholangiogram	31 (2.5)	30 (3.2)	1 (0.32)	0.003
;Intraoperative indocyanine green	93 (7.5)	9 (0.7)	84 (6.8)	< 0.001
Location	14 (1.1)	7 (0.8)		< 0.001
Ambulatory surgery center	950 (76.5)	691 (74.7)	7 (2.2)	
Community hospital	277 (22.3)	228 (24.6)	259 (82.2)	
Academic hospital			49 (15.6)	
Surgeon utilization				
N (%)	38	22 (57.9)	16 (42.1)	
Surgical division				0.054
Abdominal transplant	4 (10.5)	3 (13.6)	1 (6.3)	
Minimally invasive	14 (36.8)	8 (36.4)	6 (37.5)	
Surgical oncology	6 (15.8)	6 (27.3)	0 (0)	
Trauma, acute and critical care	14 (36.8)	5 (22.7)	9 (56.3)	
Medical school graduation (year)	1999	1994	2001	0.019
	[1990–2006]	[1982–2003]	[1985–2007]	
Fellowship training				0.3
Yes	27 (71.1)	14 (63.6)	13 (81.3)	
No	11 (28.9)	18 (36.4)	3 (19.7)	

Values represented as n (%) or median [IQR]

### Surgeon clip utilization

A total of 38 surgeons were included in this cohort. More surgeons utilized metal clips (57.9%) compared to polymer clips. Surgeons who frequently used polymer clips graduated medical school more recently (2002 vs 1994, *p* = 0.02). Completion of fellowship training did not differ between the surgeons (*p* = 0.3) (Table 2).

### Primary and secondary outcomes

Overall, 30-day ED presentation was 8.5%, with a significantly higher rate for the polymer group (12.4% vs 7.2%, *p* = 0.005; Table 3). Median time to ED presentation was 6 days [3–13; Table 3]. In the adjusted analysis, there was no significant difference in ED visits by clip type; the difference in ED presentation was significantly associated with hospital location and insurance type (Table 4). Of the 105 patients who presented to the ED, 83 (79.1%, Supplemental Table 1a) were related to the index operation, with the most common complaints being abdominal pain (56.6%) and chest pain (10.8%).

**Table 3:** 30-day postoperative outcomes

Outcome	Cohort	Metal clip	Polymer clip	*p*
	N = 1244	N = 934	N = 310	
ED presentation	105 (8.5)	67 (7.2)	38 (12.4)	0.005
Days to ED presentation	6 [3–13]	6 [3–14]	6 [3–11]	0.705
Readmission	45 (3.6)	30 (3.2)	15 (4.9)	0.173
Days to readmission	7 [3–11]	7.5 [4–16]	5 [2–11]	0.072
Infection	13 (1.0)	7 (0.7)	6 (2.0)	0.072
Fluid collection on cross-sectional imaging	10 (0.8)	6 (0.6)	4 (1.3)	0.261
Required intervention	4 (40)	2 (33.3)	2 (50)	
ERCP	9 (0.7)	6 (0.6)	3 (1.0)	0.698
Retained stone	6 (66.7)	5 (83.3)	1 (33.3)	
Cystic leak (with or without retained stone)	3 (33.3)	1(16.7)	2 (66.7)	
Death	1 (0.1)	1 (0.1)	0 (0)	0.566

Values represented as n (%) or median [IQR]

*ED* emergency department, *ERCP* endoscopic retrograde cholangiopancreatography, *IQR* interquartile range p < 0.05

**Table 4:** Predictors of ED visit within 30 days of elective laparoscopic cholecystectomy

Variable	Model	
	Odds Ratio (95% Confidence Interval	P-Value
*Clip type*		
Metal (Reference)	–	–
Polymer	1.30 (0.77, 2.19)	0.32
*Location of Procedure*		
Academic Hospital (Reference)	–	–
Community Hospital 1	0.55 (0.32, 0.94)	0.03
Community Hospital 2	0.98 (0.56, 1.72)	0.94
*Race/Ethnicity*		
Non-Hispanic White (Reference)	–	–
Hispanic of any race	1.02 (0.43, 2.42)	0.97
Non-Hispanic Black	1.41 (0.88, 2.28)	0.16
Non-Hispanic other	0.82 (0.31, 2.14)	0.68
*Insurance*		
Commercial (Reference)	–	–
Medicaid	1.75 (0.83, 3.70)	0.14
Medicare	1.10 (0.67, 1.83)	0.7
Other*	2.78 (1.21, 6.41)	0.02
*Comorbidities*		
0 (Reference)	–	–
1	1.17 (0.66, 2.08)	0.6
2 or more	1.41 (0.81, 2.45)	0.22

^*^Represents government insurance, special programs, and self-pay

The 30-day readmission rate was similar for polymer clips compared to metal clips (4.8% vs 3.2%, *p* = 0.18, respectively), as was any infection (2.0% vs 0.7%, p = 0.072), fluid collection (1.3% vs 0.6%, *p* = 0.261), and need for ERCP (1.0% vs 0.6%, *p* = 0.698). For the 45 re-admissions (Supplemental Table 1b), 14 patients had significant abdominal pain and PO intolerance requiring further work-up or inpatient management, seven patients had gallbladder fossa fluid collections requiring possible intervention, and five patients had choledocholithiasis/biliary obstruction. The remaining re-admissions encompassed a wide range of etiologies including acute coronary syndrome, electrolyte abnormalities, respiratory infection, and sickle cell crisis. Regarding the fluid collections seen in ten patients, four were clinically significant and required intervention: one patient received antibiotics only, two patients received antibiotics and a percutaneous drain, and one patient required antibiotics, a percutaneous drain, and a subsequent ERCP. Indications for ERCP included cystic duct leak (33.3%) and concern for retained stones (66.7%). Overall infection rates included deep organ space: 5(0.40%), superficial surgical site: 3(0.24%), gastrointestinal: 2(0.16%), pneumonia: 2(0.16%), and urinary tract infection: 1(0.08%). No bile duct injuries occurred during this study. One patient in the metal clip group died 22 days after surgery from acute liver failure.

### Cost of clip type

The total, unadjusted cost for a pack of six polymer clips was $48.00, with an inflation-adjusted cost of $56.40. The total, unadjusted cost for metal clips was $112.50, with an inflation-adjusted cost of $131.30.

## Discussion

This study of patients undergoing elective laparoscopic cholecystectomy demonstrates that the use of non-absorbable, polymer clips was equally safe and provides a minor cost savings to traditional metal clips. Of note, the rate of ED visits was higher in the polymer clip group with comparable readmission rates. On adjusted analysis, clip type was not significantly associated with 30-day ED visits, and the hospital facility type, along with insurance status, were the primary drivers of this association. Interestingly, a surgeon’s use of clip type was related to the year they graduated from medical school. These findings present an opportunity to understand more about intraoperative decisions and postoperative care associated with LC.

While there was an increased rate of ED visits in the polymer group on unadjusted analysis, most patients who presented to the ED did not require readmission (~ 40%), and readmission rates were similar between clip types. This is similar to prior work with elective LC showing an approximately 30% 30-day readmission rate, with 67% of ED presentations directly related to the surgery [11], similar to the 80% seen here. Adjusted analysis demonstrated an association between ED presentation based on insurance payor and status, as well as care at a particular community hospital. Regarding insurance status, there was an association between increased ED visits for patients who are self-pay and/or receiving special assistance from the hospital. Additional reasons for increase ED visits at an academic hospital may be due to the involvement of trainees or patients with lack of established care or limited accessibility to outpatient care. Pain, wound care issues, and medication refills were common and preventable reasons for ED visits. There is an opportunity to improve the postoperative care pathway for LC patients to reduce preventable ED utilization. A surgical complication can average upwards of $11,000 and providing value includes a reduction of both complication and cost [14].

Our study demonstrated that relatively few complications occurred after elective LC, regardless of clip type. While prior studies have explored outcomes of metal compared to absorbable polymer clips, only two previous studies have directly investigated clinical outcomes with non-absorbable polymer clips. Madhavan et al. (2021) found no difference in complication rates but a significantly shorter hospital stay for the polymer group [5]. In addition, a benefit of the locking mechanism on the polymer clip was related to a decreased rate of clip failure compared to metallic clips. Poillucci et al. (2021) found an improved complication profile at seven days with a similarly reduced hospital LOS for the polymer group, though the overall 30-day complication rate was similar for both groups at 9.7% [15]. Even though there are a few reports of migration, abscess, and ulcer formation [16–20], we did not experience any of these rare complications. This study overall supports a similar complication profile between clip types.

This study also demonstrated an average savings of $75 per device when polymer clips were used compared to traditional metal clips. This is limited to the six pack of polymer clips compared to an automatic clip applier with 15–20 metal clips. From our experience, the six pack of polymer clips also results in less plastic and disposable waste than the automatic clip applier. While there have been no randomized controlled trials in LC, prospective studies and RCTs for appendiceal closure with polymer clips during appendectomy have shown an associated cost reduction [21, 22]. The clip type is one component of healthcare spending that includes operative time, surgical approach, disposables and healthcare utilization. Reviewing cost with surgeons engages providers in the process of value-based care and engagement in cost containment may alter preference for disposables.

Given significant variation in pricing, local practice patterns, and a meta-analysis demonstrating that upwards of 80% of surgeons use metal clips, we sought to better understand surgeon utilization [4]. Within our hospital system, surgeon use of clip type was associated with both specialty and years in practice. Interestingly, recently graduated surgeons were most likely to use polymer clips, although there was no impact based on fellowship training. Similarly, surgeons using polymer clips were more likely to use intraoperative indocyanine green (ICG) and less likely to perform an IOC. Provider variation was not prevalent, as surgeons predominantly use one clip type during their cases and rarely switch (< 5%). While effectiveness, ease of use, and clinical safety are often primary drivers for surgeons to adopt new technology, the lack of high-level data in this space does not provide a strong impetus to switch clip types for surgeons who have developed a preference [23, 24]. Future studies should investigate individual surgeon factors and attitudes toward new technology with an emphasis on patient outcomes and cost.

This study is not without limitations, including those inherent to the retrospective nature, non-randomized design, and associated chart review. All cases were posted as elective, and outcomes may certainly differ from more urgent operations. Comorbidities were binned by overall number present rather than severity. The primary outcome was rates of presentation to the ED, rather than a comparison of direct surgery-related complications and characterization with the Clavien-Dindo scale. Additionally, this study spans multiple hospital sites that serve communities with various levels of neighborhood deprivation. Further, intraoperative or image-guided characterization of the size of the cystic duct was not recorded. Finally, healthcare dollars are spent on direct and indirect care, while medical devices contribute to a minor, but modifiable, portion of total cost.

Overall, this study demonstrates similar efficacy and outcomes between metal and polymer clips, favoring non-absorbable locking polymer clips for a minor cost-savings. As cholecystectomy is one of the most commonly performed operations worldwide, broader adoption could lead to a substantial savings. These findings provide ground for future prospective studies, in an effort to optimize patient outcomes and healthcare costs to best serve our patients undergoing LC.

## Supplementary Information

Below is the link to the electronic supplementary material.

Supplementary file1 (DOCX 36 KB)
