# Supplementary material for: Impact of metal vs non-absorbable, polymer clips during laparoscopic cholecystectomy
Source: Surg Endosc. 2025 Feb 12;39(4):2288–95. doi: 10.1007/s00464-025-11559-x (PMC11933203; doi:10.1007/s00464-025-11559-x)
Supplement: Supplementary file 1 — Supplementary file1 (DOCX 36 KB) [file 464_2025_11559_MOESM1_ESM.docx]

**Supplemental Table I.** (a) Reasons for 30-day ED presentations after elective laparoscopic cholecystectomy by hospital and (b) reason for 30-day readmission.

(a)

| Presenting symptom | N = 105 | % |
| --- | --- | --- |
| Related | 83 | 79.1 |
| Abdominal pain | 48 | 57.8 |
| Chest pain | 9 | 10.8 |
| Shortness of breath | 7 | 8.4 |
| Wound care, bleeding, infection | 5 | 6.0 |
| Urinary retention, urinary tract infection | 4 | 4.8 |
| Nausea | 3 | 3.6 |
| Hepatitis | 2 | 2.4 |
| Musculoskeletal (incision pain) | 2 | 2.4 |
| Pancreatitis | 2 | 2.4 |
| Medication refill (opioids) | 1 | 1.2 |
| Unrelated* | 22 | 20.9 |

*Reasons for unrelated ED visits include colitis, headache, hypokalemia from diuretic, pelvic pain, psychiatric crisis, sickle cell crisis, ureteral stone, trauma, etc.

(b)

| Condition requiring readmission | N = 45 | % |
| --- | --- | --- |
| Abdominal pain, nausea, emesis | 14 | 31.1 |
| Fluid collection in gallbladder fossa | 7 | 15.6 |
| Other, unrelated | 7 | 15.6 |
| Choledocholithiasis, biliary obstruction | 5 | 11.1 |
| Acute coronary syndrome, arrythmia | 3 | 6.7 |
| Hepatic failure, metabolic encephalopathy | 3 | 6.7 |
| Cerebrovascular accident | 1 | 2.2 |
| Pancreatitis | 1 | 2.2 |
| Pneumonia | 1 | 2.2 |
| Surgical site infection | 1 | 2.2 |
| Urinary tract infection | 1 | 2.2 |

*Reasons for other include sickle cell crisis, electrolyte abnormalities, ureteral stone, endoscopy, etc.w
